# Arthroscopic Osteosynthesis for the Treatment of Coronoid Process Fractures: A Case Series

**DOI:** 10.1155/2018/8512963

**Published:** 2018-06-19

**Authors:** Noriaki Shimada, Katsuhiko ShirakiIt, Kazuo Saita

**Affiliations:** Department of Orthopaedic Surgery, Saitama Medical Center, Saitama Medical University, 1981 Kamoda, Kawagoe, Saitama 350-8550, Japan

## Abstract

The treatment strategy and surgical indication for coronoid process fractures are not clear. Many methods of surgery have been described. We report on the use of arthroscopic osteosynthesis for this type of fracture. This method is minimally invasive and effective for confirming the reduction, and it is advantageous for postoperative pain and early recovery after surgery.

## 1. Introduction

Fractures of the ulna coronoid process are relatively rare and are reported to occur in 2%–10% of patients who have a dislocated elbow [1]. The ulna coronoid process is of critical importance to the elbow stability [2, 3]; however, the treatment strategy and surgical indication are not clear. Because the fracture site is deep within the tissue, open reduction internal fixation of the fracture is invasive and difficult. We report on the use of arthroscopic osteosynthesis for the fracture. The arthroscopic approach offers many advantages, including a smaller incision, easy access to the joint and fracture site, less soft tissue dissection, and reduced postoperative pain. This method is minimally invasive and effective for confirming the reduction.

The purpose of this report was to present the technique of arthroscopic osteosynthesis in the coronoid process fractures and the clinical results of patients who underwent this surgery.

## 2. Case Presentation

Informed consent was obtained from all patients who took part in this report.

### 2.1. Case 1

**Figure 1:**
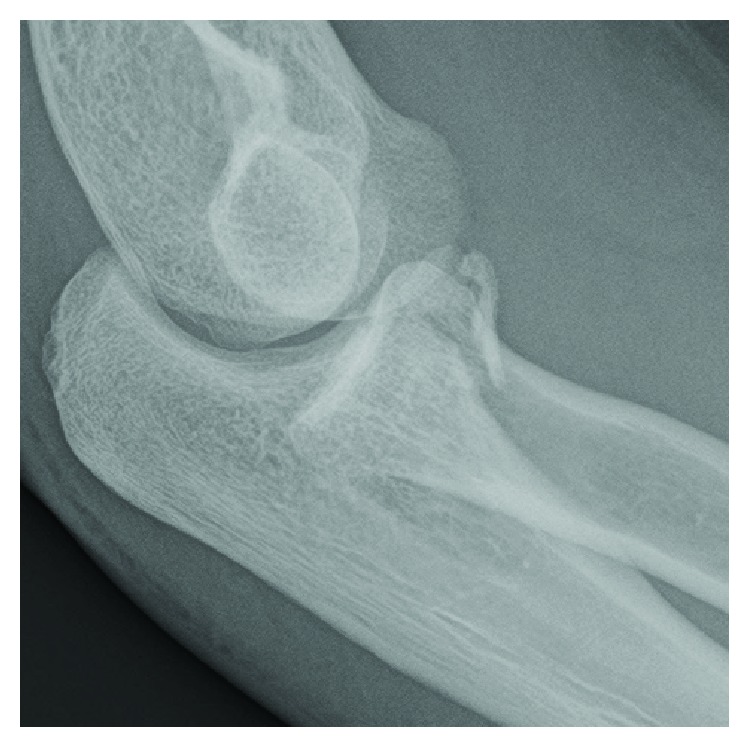
Case 1: radiograph demonstrates a type 2 coronoid fracture.

A 39-year-old man fell and injured himself whilst walking on the road. Upon physical examination, he complained of severe pain in his right elbow, which showed swelling and tenderness. Because of the pain, the range of motion of the elbow joint was limited to 30°–50°. Radiographs revealed a fracture of the ulna coronoid process, which was displaced by about 4 mm (Figure 1). He was diagnosed with a coronoid process fracture, Regan-Morrey type 2. He was young, highly active, and wanted an early return to normal life; therefore, we performed a minimally invasive arthroscopic osteosynthesis 7 days after the injury.

**Figure 2:**
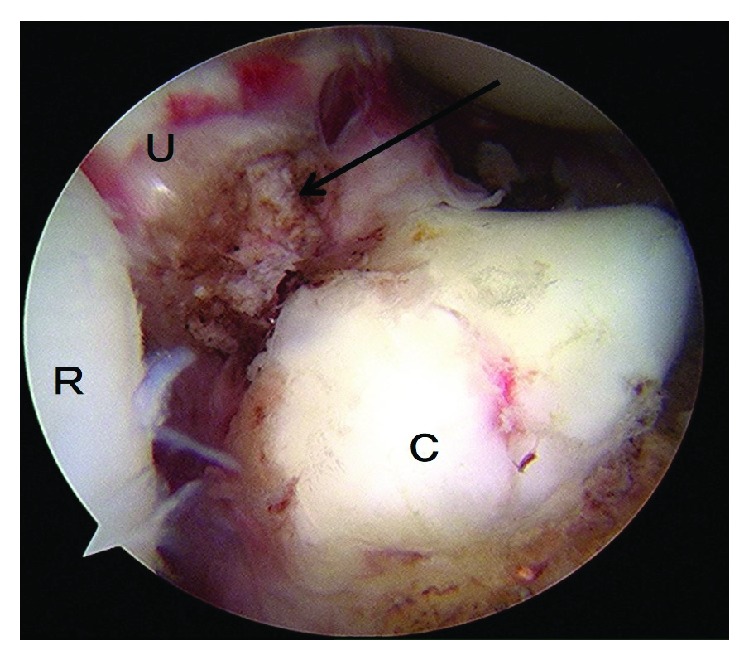
Case 1: arthroscopic photograph shows the fracture site (arrow). R: radial head; U: ulna (fracture bed); C: coronoid process.

**Figure 3:**
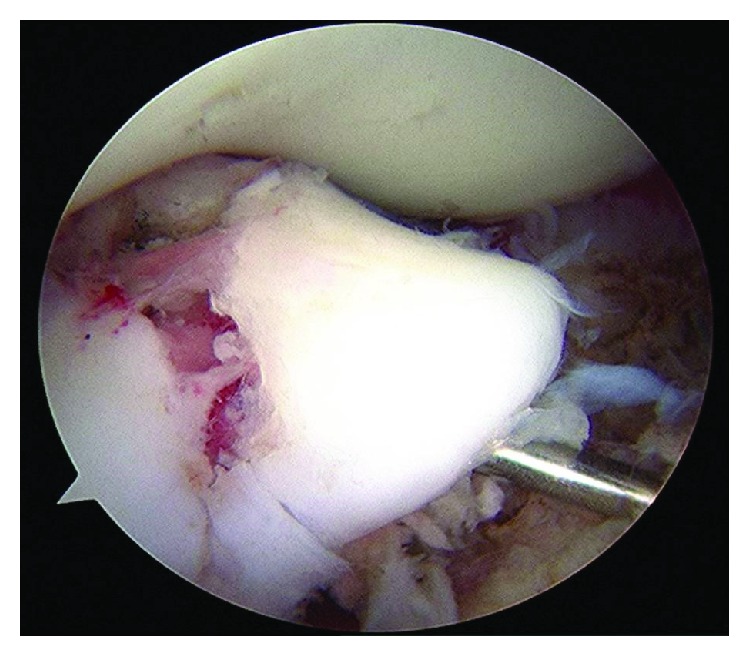
Case 1: a guide pin is inserted from the posterior side.

**Figure 4:**
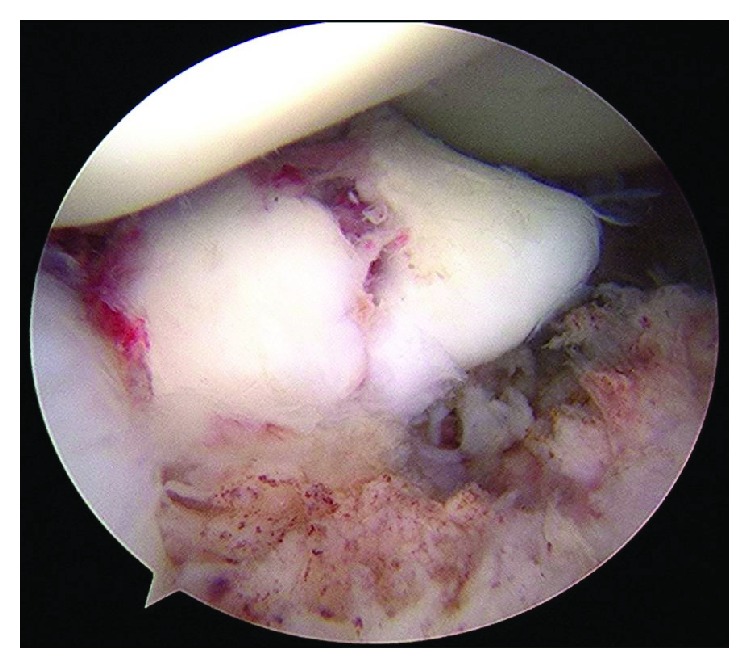
Case 1: arthroscopic photograph shows a good reduction position after fixation by a CCS and K-wire.

**Figure 5:**
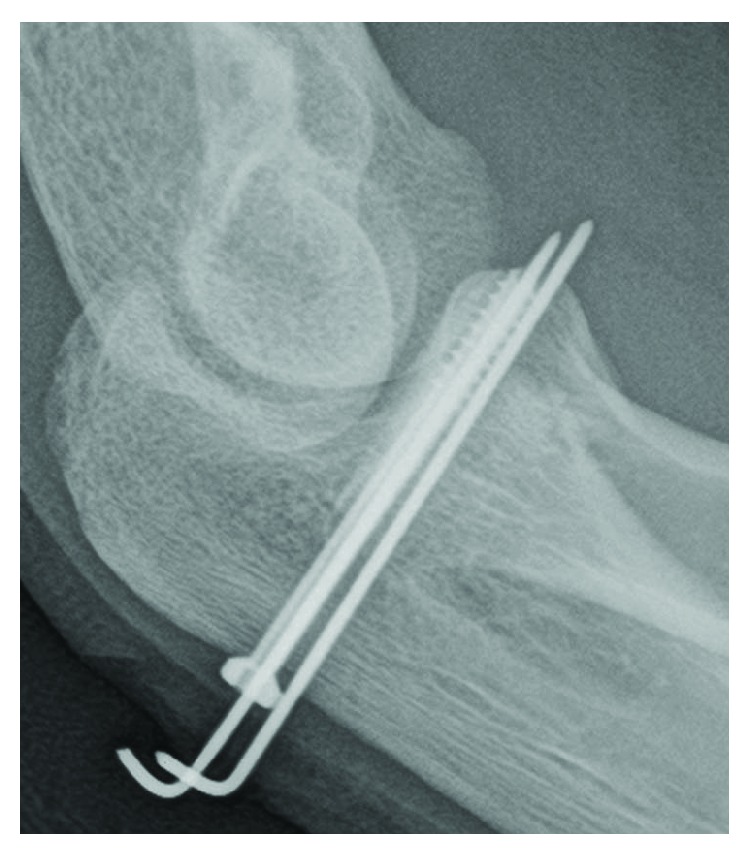
Case 1: postoperative radiograph shows a good reduction position.

**Figure 6:**
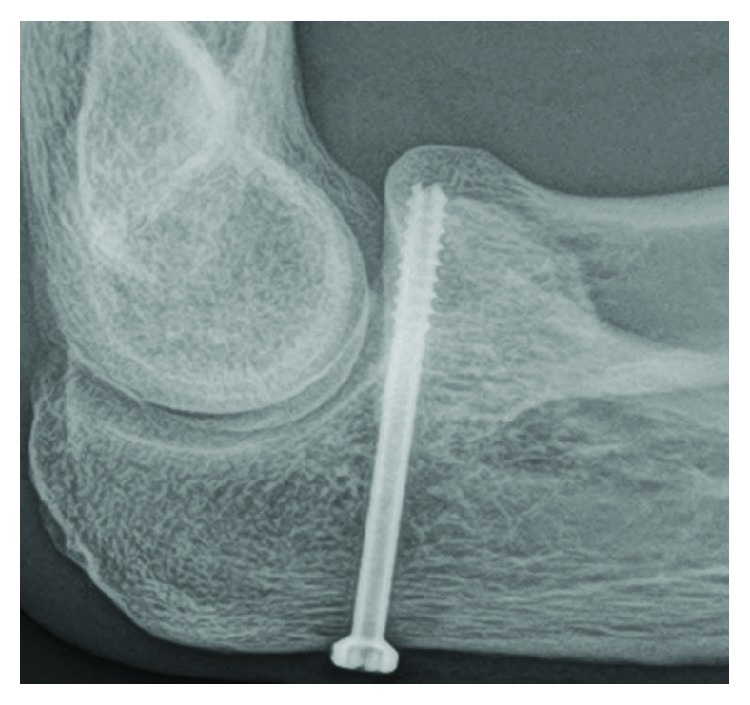
Case 1: radiograph 4 months after the operation shows bone union.

Surgery was performed under general anaesthesia with the patient in the prone position. We confirmed the fracture site through the anterolateral portal (Figure 2). A shaver and radiofrequency probe were used to remove any clots and debris. The radiofrequency probe allowed us to reduce the amount of bone fragment entering the fracture bed. Under fluoroscopic control, a guide pin for a cannulated cancellous screw (CCS) was inserted from the posterior ulnar shaft into the coronoid fragment (Figure 3). We fixed the fracture with a 3.5 mm CCS and two 1.5 mm K-wires, whilst confirming the anatomical reduction arthroscopically (Figure 4). After fixation, we confirmed a good reduction position and sufficient stability upon examination. We also confirmed a reduction by radiographs (Figure 5). Postoperatively, he wore an immobilizing splint for 1 week and then began physical therapy to increase the range of motion. After 3 weeks, the K-wires were removed, and bone union was achieved after 4 months (Figure 6). At 1 year postoperatively, he had no pain and a physical examination revealed a complete range of motion.

### 2.2. Case 2

**Figure 7:**
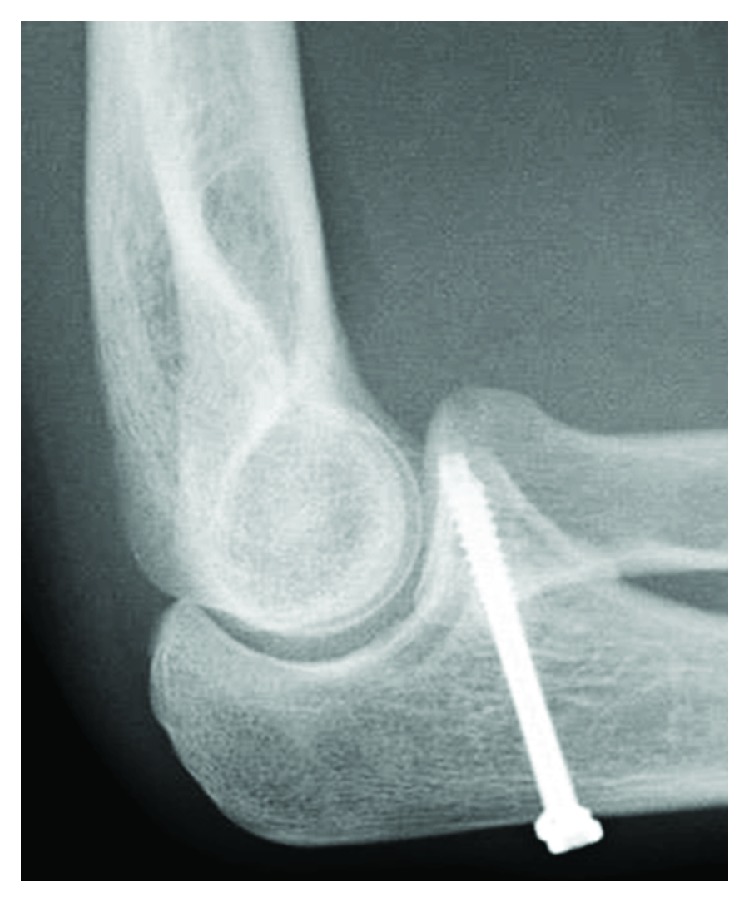
Case 2: postoperative radiograph shows fixation by two CCSs.

A 29-year-old woman fell and injured herself whilst walking down the stairs. She was also diagnosed with a coronoid process fracture, Regan-Morrey type 2. We performed arthroscopic osteosynthesis 5 days after the injury in almost the same way as described in Case 1, using two 3.5 mm CCSs (Figure 7). Postoperatively, she also had a good clinical course; she had no pain and had full range of motion of the elbow joint 1 year after surgery.

### 2.3. Case 3

**Figure 8:**
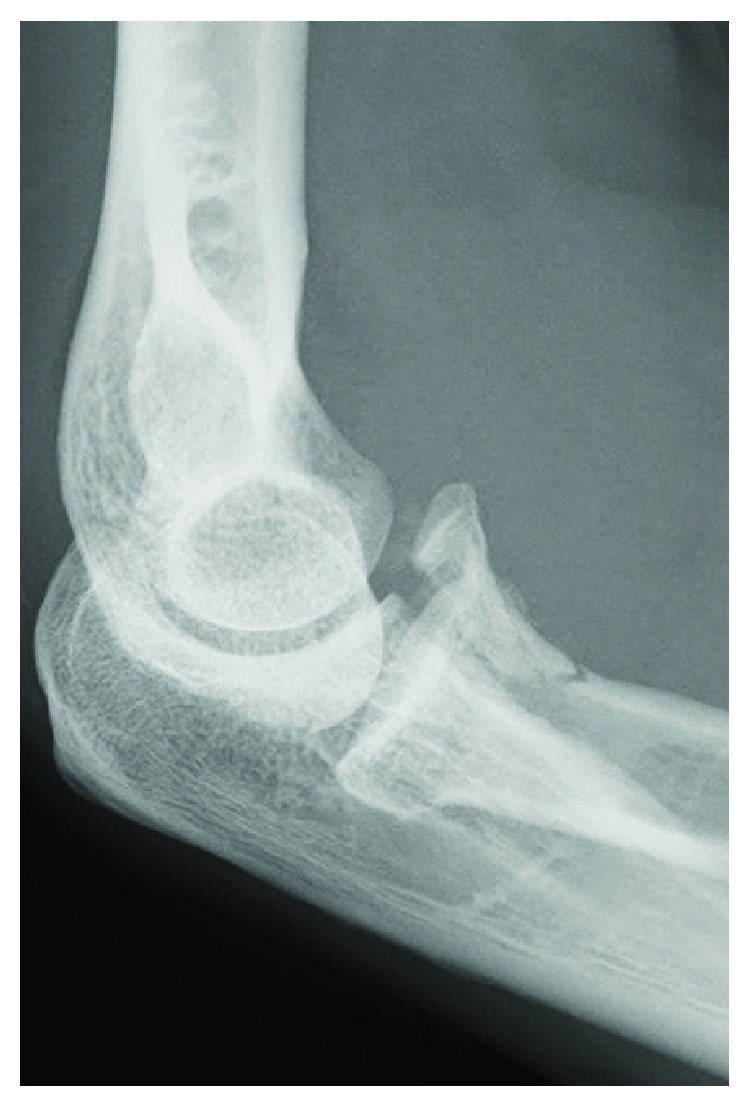
Case 3: radiograph demonstrates a type 2 coronoid fracture.

**Figure 9:**
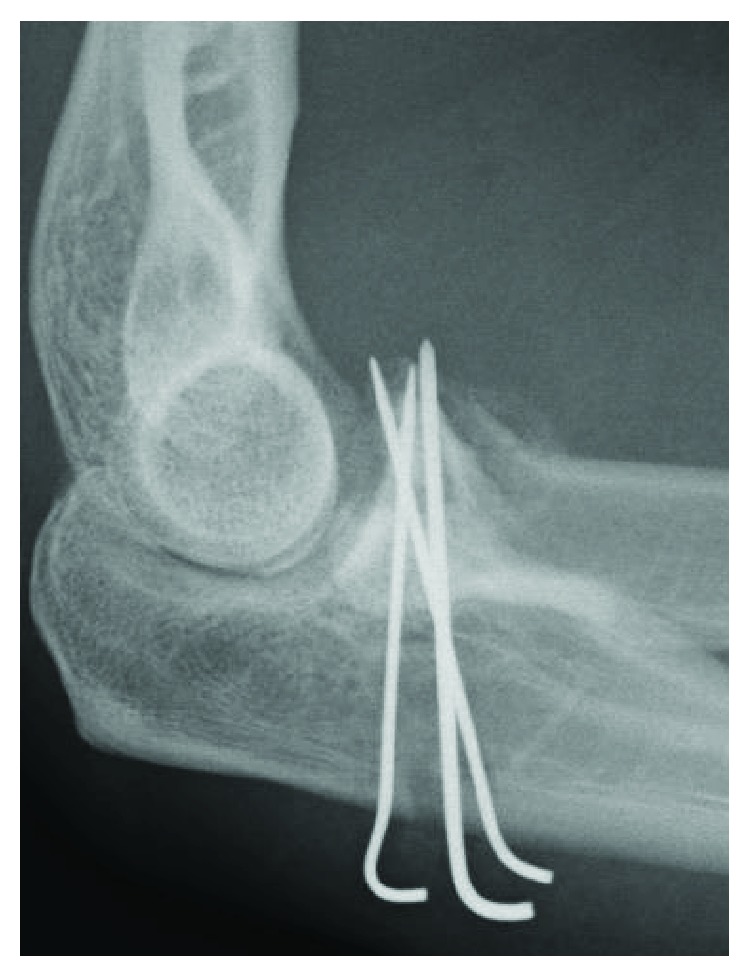
Case 3: postoperative radiograph shows fixation by three K-wires.

**Figure 10:**
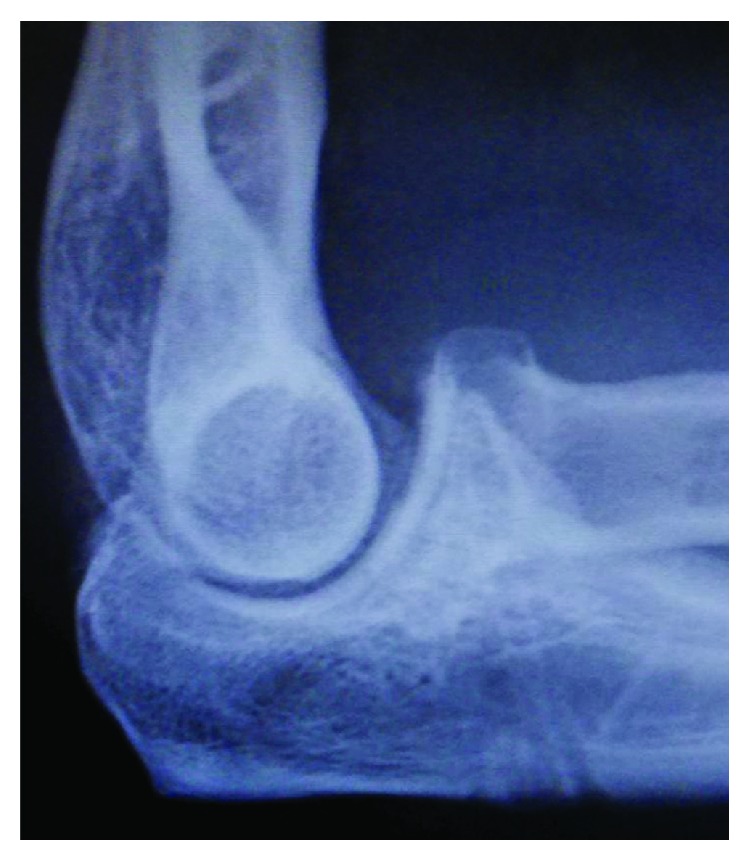
Case 3: radiograph 8 months after the operation shows bone union and removal of the K-wire.

A 40-year-old man fell and injured himself whilst walking. He was also diagnosed with a coronoid process fracture, Regan-Morrey type 2 (Figure 8). We performed arthroscopic osteosynthesis 10 days after the injury in almost the same way as described in Case 1. However, the fracture was too comminuted to be fixed by CCS, so we performed osteosynthesis using three K-wires (Figure 9). Postoperatively, he wore an immobilizing splint for 2 weeks and then began physical therapy to improve the elbow range of motion. After 4 months, bone union was confirmed, and after 8 months, the K-wires were removed (Figure 10). A follow-up at 10 months showed that he had no pain, and a physical examination revealed complete range of motion, indicating a good clinical course.

## 3. Discussion

The ulna coronoid process is of critical importance to elbow stability, and strong internal fixation is required for this fracture type [2, 3]. It is generally agreed that Regan-Morrey type 1 fractures are recommended with a nonoperative treatment, whereas type 3 fractures are recognized to require osteosynthesis to avoid recurrent elbow instability [2]. However, opinions are divided on the treatment policy for type 2 fractures [2, 4–9]. Many open methods employing K-wires, screws, soft wires, mini plates, sutures, and others have been described [10–14]. However, these methods require surgery close to the neurovascular bundle; it is quite invasive as the fracture extends deep into the tissue, making it difficult to secure a visual field. Therefore, there are concerns about the occurrence of neurovascular injury, postoperative pain, contracture due to large soft tissue invasion, and insufficient reduction and internal fixation due to the poor visual field.

In accordance with the progress of arthroscopic techniques, arthroscopic osteosynthesis for coronoid fractures has been described [14–18]. In arthroscopic osteosynthesis, we can avoid an extensive surgical approach and reach the lesion directly. Therefore, soft tissue invasion is minimal and we can confirm deep layer lesions in detail using the arthroscope monitor. Another advantage of arthroscopic osteosynthesis is that the method of fixing with CCS from the posterior ulnar shaft is simple and it provides a strong fixation. We have successfully performed arthroscopic osteosynthesis in three patients, all of which had good clinical results. In Case 3, we were unable to use CCS, so we performed internal fixation only using K-wires. However, it still had a good clinical result, as in Case 1 and Case 2. We think our method is simple and able to get successful result.

## 4. Conclusion

Arthroscopic osteosynthesis for ulna coronoid fractures is minimally invasive and is considered to be advantageous for postoperative pain and early recovery.
